# Transcriptome regulation and chromatin occupancy by E2F3 and MYC in mice

**DOI:** 10.1038/sdata.2016.8

**Published:** 2016-02-16

**Authors:** Xing Tang, Huayang Liu, Arunima Srivastava, Thierry Pécot, Zhong Chen, Qianben Wang, Kun Huang, Maria Teresa Sáenz-Robles, Paul Cantalupo, James Pipas, Gustavo Leone

**Affiliations:** 1 Department of Molecular Virology, Immunology and Medical Genetics, College of Medicine, Columbus, Ohio 43210, USA; 2 Department of Molecular Genetics, College of Biological Sciences, Columbus, Ohio 43210, USA; 3 Comprehensive Cancer Center, Columbus, Ohio 43210, USA; 4 Department of Molecular and Cellular Biochemistry, Columbus, Ohio 43210, USA; 5 Department of Biomedical Informatics, The Ohio State University, Columbus, Ohio 43210, USA; 6 Department of Biological Sciences, University of Pittsburgh, Pittsburgh, Pennsylvania 15260, USA

**Keywords:** Mitosis, Transcriptional regulatory elements, Cell proliferation, Microarray analysis, Chromatin immunoprecipitation

## Abstract

E2F3 and MYC are transcription factors that control cellular proliferation. To study their mechanism of action in the context of a regenerating tissue, we isolated both proliferating (crypts) and non-dividing (villi) cells from wild-type and *Rb* depleted small intestines of mice and performed ChIP-exo-seq (chromatin immunoprecipitation combined with lambda exonuclease digestion followed by high-throughput sequencing). The genome-wide chromatin occupancy of E2F3 and MYC was determined by mapping sequence reads to the genome and predicting preferred binding sites (peaks). Binding sites could be accurately identified within small regions of only 24 bp-28 bp long, highlighting the precision to which binding peaks can be identified by ChIP-exo-seq. Forty randomly selected E2F3- and MYC-specific binding sites were validated by ChIP-PCR. In addition, we also presented gene expression data sets from wild type, *Rb-, E2f3-* and *Myc*-depleted crypts and villi within this manuscript. These represent comprehensive and validated datasets that can be integrated to identify putative direct targets of E2F3 and MYC involved in the control of cellular proliferation in normal and *Rb*-deficient small intestines.

## Background & Summary

E2F is a family of transcription factors that links up-stream proliferation signals with the timely expression of many downstream cell cycle regulated genes^1^. Among the E2F members, E2F3 is known as a major transcriptional activator that can be inhibited when in complex with RB. During late G1 phase of the cell cycle, CDK mediated phosphorylation of RB leads to the release of E2F3 from RB-E2F3 complexes and the activation of the G1–S transcriptional program, resulting in entry of cells into S phase^1^. MYC is another important transcriptional regulator of the cell cycle^2^. It’s an oncogene with a broad functional spectrum, which includes the control of cellular proliferation, cell survival, cell growth and metabolism^3^. Despite significant functional overlap between E2F and MYC in the control of cell cycle regulation, the mechanisms linking these two critical transcriptional programs are poorly understood.

**Figure 1:**
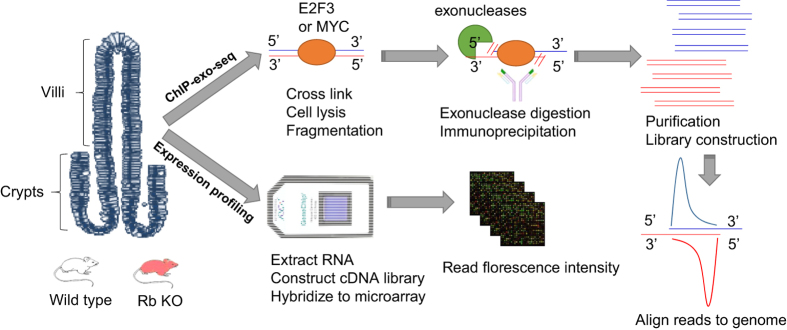
Flow chart of experimental design. Crypts and villi collected from wild type/*Rb* deficient small intestine of mouse are collected for ChIP-exo-seq and microarray experiment separately.

The small intestine has been utilized as a model system to study normal cellular proliferation and cancer. A unique characteristic of the small intestine is that the continuous regeneration of the entire intestinal track is neatly organized in a defined pouch-like invagination called the crypt^4^. At the base of crypts, pluripotent stem cells asymmetrically divide to generate a new stem cell and a transient amplifying progenitor cell. Progenitor cells undergo multiple rounds of cell division to form a transient amplifying zone. As progenitor cells migrate towards the finger-like protrusions called villi, they exit the cell cycle and differentiate^4^. Thus, proliferating progenitor cells and differentiated enterocytes are located in distinct spatial regions of the intestinal track as depicted in Fig. 1. We chose to evaluate E2F3 and MYC chromatin occupancy in cells of the small intestine in order to better define how these two factors regulate the cell cycle *in vivo*.

ChIP-seq is a widely used technique to profile chromatin occupancy of transcription factors on a genome wide scale. Briefly, chromatin is released from lysed cells and sheared to smaller fragments and specific antibodies are used to immunoprecipitate protein-DNA complexes. Then, the enriched DNA fragments are purified, tagged, amplified and sequenced using next-generation sequencing technology. Finally, genomic regions with enriched read alignments are detected as predicted protein binding sites, which usually span hundreds of base pairs^5,6^. Recently, an improved technique to better define chromatin binding regions was developed, ChIP-exo-seq^7,8^. The key innovation of this technique is that after ChIP, the lambda exonuclease is used to digest DNA fragments starting from the exposed 5′ end and stopping at the protein-DNA boundary. After library preparation and deep sequencing, the 5′ end of the reads obtained are highly concentrated on the protein-DNA boundary, providing better positional resolution and accuracy of the predicted protein binding sites^7,8^.

Both ChIP-exo-seq experiments and expression microarray experiments were conducted with enriched crypt and villus fractions obtained from the small intestine of mice (Table 1, available online only). For ChIP-exo-seq experiments, E2F3 and MYC chromatin binding was analyzed in parallel in crypts and villi derived from wild type and *Rb* deficient small intestines. An average of 70 million to 120 million reads were generated for each ChIP-exo-seq experiment (Table 2). For gene expression assays, we used an Affymetrix microarray platform (Mouse Genome 430 2.0 Array) to profile mRNA levels in crypts and villi derived from various genetically modified mice with respect to *E2f, Myc* and *Rb* deficiency (Table 1, available online only). The intersection between ChIP-exo-seq and mRNA expression data sets are identified as putative direct targets of MYC and E2F3. Comparison of putative MYC and E2F3 target genes revealed unique and overlapping sets of targets, suggesting distinct and synergistic roles for MYC and E2F3 in the control of gene expression in the small intestine. Our related work recently published in *Nature Cell Biology* used these molecular approaches to address different biological questions related to the control of cellular proliferation *in vivo*^9^. In this Data Descriptor, we provide additional information aimed to help other investigators interpret and use these data sets for their own research.

## Methods

### Mouse usage

The description of the mouse strains used and how they were maintained is described in detail in the related research manuscript^9^. Briefly, mice were housed at the Ohio State University ventilated animal vivarium under standard conditions (temperature at 22 °C and 12 h light/12 h dark cycle per day). Experimental manipulation of animals was approved by Institutional Animal Care and Use Committee at the Ohio State University. Mouse strains were bred and maintained in a mixed genetic background (C57BL/6×129×FVB/N). Both genders of mice were utilized in the studies. To induce the expression of Cre recombinase and deletion of alleles flanked by *loxP* sequences, β-naphthoflavone (Sigma-Aldrich; N3633) dissolved in corn oil (Sigma-Aldrich; C8267) was intraperitoneally injected into 2-month old mice at the dosage of 80 mg kg^−1^ body weight^10^. Five injections were performed within 30 h (9am-3pm-9am on first day and 9am-3pm on second day).

### Mouse tissue collection

Immediately after euthanizing mice, the small intestine was dissected and mesentery and adipose tissues were removed. The intestine was then cut open along their cephalocaudal axis and gently washed in phosphate buffered saline (PBS) to remove undigested food chow. The tissue used for RNA isolation was collected from a 10 cm portion of the intestine proximal to the stomach. For ChIP, the intestine was divided into three parts of equal length and the first 1/3 section proximal to the stomach was used. After cleaning with PBS, the tissue was incubated in 25 ml PBS containing 0.5 mM ethylenediaminetetraacetic acid (EDTA) and 1 mM dithiothreitol (DTT). After incubation for 30 min at room temperature, villus fractions were collected by multiple rounds of gently shaking the tissue, decanting tissue materials in suspension into multiple tubes containing 10 ml ice-cold PBS with 1 mM DTT. After most villi were collected, the remaining tissue was incubated in 25 ml PBS with 0.9 mM EDTA and 1 mM DTT. The crypt fractions were collected by shaking the tissue in multiple tubes containing 10 ml ice-cold PBS with 1 mM DTT until complete separation of epithelial cells from mesenchymal tissues. Crypt-enriched fractions were filtered using 70 μm cell strainer (Fisher Scientific; 22363548) to remove potential contamination of broken pieces of villi with larger sizes. After centrifuge at 335×g for 5 min at 4 °C, the resulting villus-enriched or crypt-enriched tissue pellets were combined and washed in ice-cold PBS. The tissues were then precipitated by centrifuge at 335×g for 5 min at 4 °C. For RNA isolation and ChIP assays, the cell pellets were processed as described below.

### RNA isolation

Total RNA from purified villi/crypts was isolated using TRIzol reagent following manufacturer’s protocol and further cleaned up with RNeasy Mini Kit (Qiagen; 74104). RNA integrity was assessed using Agilent 2100 Bioanalyzer Instruments. Global RNA expression levels were profiled using Affymetrix GeneChip Mouse Genome 430 2.0 Array at the Ohio State University Shared Resources (http://cancer.osu.edu/research-and-education/shared-resources/genomics/services).

### ChIP

Freshly isolated crypts/villi were crosslinked in PBS containing 1% formaldehyde at 37 °C for 15 min on a rotator. The crosslinking reaction was terminated by incubation with 0.125 M glycine at 37 °C for 5 min on a rotator. The cell pellets were then washed in ice-cold PBS, followed by cytosolic lysis and nuclear lysis steps (cytosolic lysis buffer: 5 mM PIPES (pH 8.0), 85 mM KCl, 0.5% NP-40; nuclear lysis buffer: 50 mM Tris (pH 8.0), 10 mM EDTA, 1% SDS). The released chromatin was then subject to sonication to generate DNA fragments mainly with 100-300 bp sizes. The fragmented chromatin was diluted with 9 volumes of IP dilution buffer (16.7 mM Tris (pH 8.0), 167 mM NaCl, 1.2 mM EDTA, 1.1% Triton X-100). To minimize non-specific pull-down, the chromatin was incubated with Protein G Plus/Protein A beads (Calbiochem; IP05) at 4 °C for 1 h on a rotator prior to adding antibodies. Antibodies used in the study were E2F3 (Santa Cruz; sc-878 X), MYC (Santa Cruz; sc-764 X) and normal rabbit IgG (Santa Cruz; sc-2027). The chromatin was incubated with antibodies overnight at 4 °C on a rotator, followed by incubation with Protein G Plus/Protein A beads (Calbiochem; IP05) at 4 °C for 2 h on a rotator. The chromatin-antibody-bead complex was then washed twice with each of the following buffers: low-salt buffer (20 mM Tris (pH 8.0), 2 mM EDTA, 1% Triton X-100, 0.1% SDS and 150 mM NaCl), high-salt buffer (20 mM Tris (pH 8.0), 2 mM EDTA, 1% Triton X-100, 0.1% SDS and 500 mM NaCl), LiCl buffer (10 mM Tris (pH 8.0), 1 mM EDTA, 250 mM LiCl, 1% NP-40 and 1% deoxycholic acid) and Tris-EDTA buffer (pH 8.0). Note that all the steps described above were performed in the presence of proteinase inhibitors. The Protein G Plus/Protein A beads were incubated with 1% SDS plus 0.1M NaHCO_3_ (900 rpm vortex) to retrieve the immunoprecipitated chromatin. The chromatin was then reverse-crosslinked by incubation with 0.5M NaCl and 0.1 mg/ml RNase A overnight at 65 °C (including non-immunoprecipitated inputs), followed by 0.1 mg/ml proteinase K treatment at 50 °C for 1 h. The DNA was purified with Qiagen kits (QIAquick Purification Kit (Qiagen; 28104). Quantitative PCR was performed using SYBR Green master mix (Bio-Rad; 170-8884) with Applied Biosystems StepOnePlus Real-Time PCR System.

### ChIP-exo-seq

For ChIP-exo-seq experiments, crypts were collected and pooled from 32 wild type mice and 27 *Rb KO* mice and villi were collected from 7 wild type mice and 7 *Rb KO* mice. The subsequent steps, including crosslinking, cytosolic and nuclear lysis, sonication, dilution with IP dilution buffer, pre-clean and immunoprecipitation (incubation with antibodies and beads) were performed as described above in the ChIP section, except Pierce Protein A/G Magnetic Beads (Thermo Scientific; 88802) were used instead of Protein G Plus/Protein A beads. Prior to adding antibodies, the pooled chromatin derived from each genetic/tissue compartment was divided into two for E2F3- and MYC-ChIP, respectively. After generating the chromatin-antibody-bead complex, the library construction steps including on-bead enzymatic reactions (end polishing, P7 exo-adapter ligation, nick repair, λ-exonuclease digestion, RecJ_f_ exonuclease digestion), elution and reverse-crosslinking, primer extension and P5 exo-adaptor ligation were performed as described^11^. The resulting DNA was enriched by 13 cycles of PCR using NEBNext High-Fidelity PCR Master Mix (NEB; M0541S). The DNA concentration and DNA size distribution of libraries were profiled using Agilent High Sensitivity DNA Kit. Cluster generation on Illumina cBot and single-end high throughput sequencing on Illumina HiSeq 2500 platform were performed at the Ohio State University Shared Resources (http://cancer.osu.edu/research-and-education/shared-resources/genomics/services).

### Microarray data analysis

For microarray analysis, functions from R package Affy^12^ were utilized. The ‘justRMA’ function was used to read the raw CEL files, perform background correction and normalization. The ‘exprs’ function was used to obtain the log2 transformed expression value. All parameters were set as default.

### ChIP-exo-seq data processing

After base calling, adapter trimming, and barcode demultiplexing using the sequencer manufacturer’s software, raw sequences with quality scores (Sanger/Illumin1.9) were stored in FASTQ format. The name of each sample includes the mouse genotype, tissue type and the specific antibody used. Each sample was sequenced in two lanes of an Illumina flow cell and the resulting data was combined into one FASTQ file before mapping onto the genome.

We used Bowtie2 (ref. 13) to align short reads onto the mouse genome (mm9) with -N 1 to allow only one mismatch and output alignments into SAM formatted files using -S option. Two algorithms were then used with the default significance cutoffs (GEM^7^: FDR<0.01; MACS2 (ref. 5): FDR<0.05) to detect peaks enriched on the genome. Any binding events identified by GEM that were not identified by MACS2 were filtered out by using ‘window’ command from bedtools^14^ with -w 1000. We used GEM to predict binding positions and MACS2 to filter binding events because GEM has a higher accuracy of predicting binding locations and binding motifs. To predict genes regulated by these binding events, ‘refgene_getnearestgene’ command from CisGenome^15^ was used to find the nearest gene for each binding event. We set -dt 1, -up 50,000,000 and -down 30,000,000 so that binding events located 50 M bp upstream or 30 M bp downstream of the nearest TSS were discarded. The gene annotation file we used is mm9_refFlat_sorted.txt, which was downloaded from CisGenome website. Next, we used HOMER^16^ script findMotifsGenome.pl with -size 100 to do de novo searches of enriched sequence motifs within 100 bp regions of the binding events. Finally, HOMER script annotatePeaks.pl was used to calculate read coverage over the 500 bp regions of binding events and HOMER script Homer_plot.R was used to visualize the read coverage.

### Code availability

The version of each software used in this paper are noted below.

bedtools v2.17.0

samtools 0.1.19-44428 cd

FastQC v0.11.2

bowtie2 version 2.2.1

macs2 2.0.10.20131216 (tag:beta)

GEM (version 2.4.1)

homer v4.6

IGV Version 2.3.32

All the scripts used to run tools mentioned before or process intermediate files are uploaded onto figshare (https://dx.doi.org/10.6084/m9.figshare.2059239)

## Data Records

ChIP-exo-seq data from 8 samples (4 from crypts and 4 from villi) were deposited in Gene Expression Omnibus^17^ (NCBI) under accession number GSE56008 (Data Citation 1). For each sample in GSE56008, a processed data file listing all the predicted binding sites is provided. It is a tab-separated text file in BED format with the following columns: Chromosome Name, Start Coordinate, End Coordinate, Binding Site ID, and Binding Site Score (calculated from GEM). Links to the corresponding NCBI Sequence Read Archive (SRA) accession, which deposits the raw sequencing data (Data Citation 2), are also provided.

**Figure 2:**
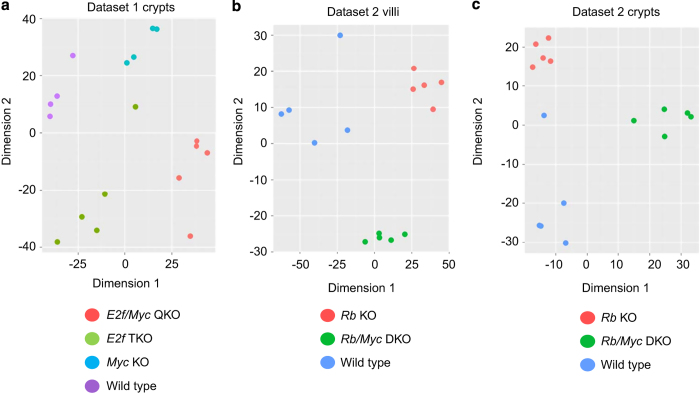
Multidimensional Scaling (MDS) plots for microarray gene expression data. (**a**) ‘Dataset 1 crypts’, (**b**) ‘Dataset 2 villi’, (**c**) ‘Dataset 2 crypts’. Samples with different genotypes are colored differently. *E2f* TKO: *E2f1–3* triple KO; *Rb/Myc* DKO: *Rb* and *Myc* double KO; *E2f/Myc* QKO: *E2f1–3* and *Myc* quadruple KO.

Microarray Dataset 1 in Fig. 2 were deposited in GEO under the accession number GSE56006 (Data Citation 3). Microarray Dataset 2 in Fig. 2 were deposited in GEO under accession number GSE56007 (Data Citation 4). Raw signal files in.CEL format are also provided for each sample. Users could freely download them and reanalyze them with R package Affy.

## Technical Validation

### ChIP-exo-seq

**Figure 3:**
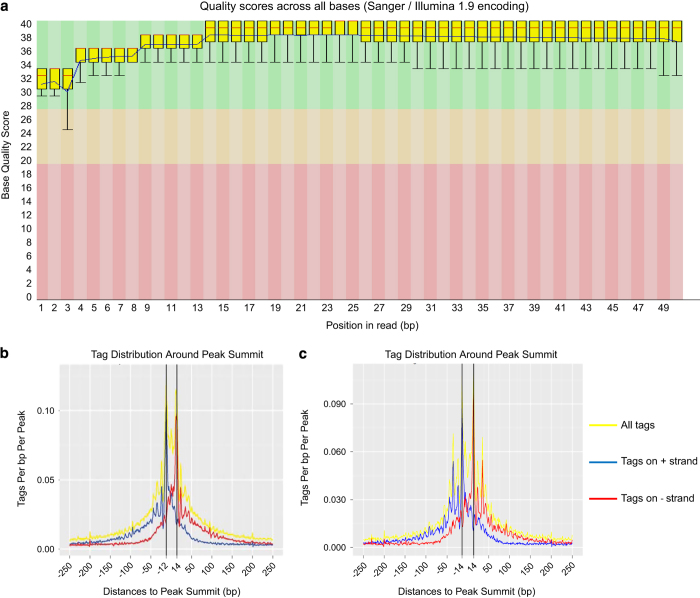
Quality control analysis of ChIP-exo-seq data sets. (**a**) Bar plot for bases quality score from a representative sample ‘RbKO villi anti-E2F3’. Tag (5 prime end of read) distribution surrounding predicted binding events on average from representative samples (**b**) ‘RbKO villi anti-E2F3’ and (**c**) ‘RbKO villi anti-MYC’. Tags from each DNA strand are shown both separately (blue: +; red:−) and combined together (yellow) on the same plot.

To survey the quality of our ChIP-exo-seq data the average quality score for each nucleotide position was determined (Sanger/Illumina1.9 format). Figure 3a shows an example of the quality score for each position (bp) in tag reads for the E2F3-specific ChIP-exo-seq data set derived from *Rb* deleted villi. In all ChIP-exo-seq experiments the quality score was determined to be above 30 (Fig. 3a). Over 90% of the sequenced reads were mapped successfully to the mouse genome (mm9), where over 60% of reads mapped to unique genome positions (Table 2). Thus, based on nucleotide position quality score and the percentage of reads mapped to the genome, we conclude that the sequencing quality of our ChIP-exo-seq data is very good.

Reads aligned to the positive and negative strands formed independent single peak summits for both E2F3 and MYC ChIP experiments. Sequences between the two summits, which were 24 bp–28 bp in length, represent the E2F3 and MYC binding sites (Fig. 3b and c). The number of reads between the two summits were >19-fold higher than in other genomic regions (Table 3). Thus, ChIP-exo-seq derived reads sharply define the protein binding sites of E2F3 and MYC on chromatin.

To validate the predicted transcription binding sites, forty randomly selected E2F3- and MYC-specific peaks were assessed by ChIP-PCR assays in *E2f3*-deficient and *Myc*-deficient tissues, respectively. All selected E2F3- and MYC-specific peaks were highly enriched in wild type tissues when compared to *E2f3*- and *Myc*-deficient tissues (see NCB paper^9^ Fig. 4b). In addition, control IgG ChIP-PCR assays showed low background binding to these selected E2F3- and MYC binding sites. Together, these control experiments highlight the highly specific nature of E2F3- and MYC chromatin profiling by ChIP-exo-seq.

We then identified transcription factor binding motifs imbedded within 100 bp region of the identified binding sites. As expected, canonical E2F binding motifs were significantly enriched in crypts and villi of both wild type and *Rb* deficient tissues. Interestingly, canonical MYC binding motifs were only enriched in *Rb* deficient crypts, highlighting the impact of *Rb* loss on MYC global chromatin binding. Detailed information of motif sequences and distribution for each experiment is listed in Supplementary Fig. 6b of the reference manuscript^9^.

### Expression profiling

Microarray gene expression data sets were first background corrected and normalized, and then the log_2_ transformed expression values were used to perform Multidimensional Scaling (MDS) analysis. As shown in Fig. 2, replicate samples within the same genetic cohort grouped together. Finally, gene expression changes between genetic cohorts were validated by real-time PCR expression assays (Figs 3b, 3c, 6d and Supplementary Fig. 3 of Liu *et al*.,^9^). Thus, we conclude that the overall reproducibility of microarray gene expression data sets is very good.

## Usage Notes

Scripts for running Bowtie2, GEM, MACS2, HOMER and scripts for processing other intermediate files have been uploaded to figshare (https://dx.doi.org/10.6084/m9.figshare.2059239). These scripts were run on a linux system. To deploy the pipeline, please download and decompress the script archives, install required software and configure them in the ‘software.conf’ file. Then copy your raw FASTQ files into the ‘rawdata’ folder and configure them in the ‘samples.conf’ file. Finally, the ‘bash run_ChIP-exo_pipeline.sh’ command is used to execute the analysis pipeline. The predicted binding regions, corresponding target genes, and enriched sequence motifs of each ChIP-exo-seq experiment will be automatically generated. Intermediate files, such as alignments in BAM format and read coverage along the genome, will also be generated and could be visualized using IGV^18^. The parameters can be adjusted by revising the corresponding scripts. This package may be modified to accommodate other species.

## Additional Information

**How to cite this article**: Tang, X. *et al*. Transcriptome regulation and chromatin occupancy by E2F3 and MYC in mice. *Sci. Data* 3:160008 doi: 10.1038/sdata.2016.8 (2016).

## Supplementary Material



## Figures and Tables

**Table 1:** Metadata for experiments.

**Subjects**	**Protocol 1**	**Protocol 2**	**Protocol 3**	**Protocol 4**	**Data**
2 month old adult mice	wild type intestines	villi tissue collection	RNA extraction	Affymetrix GeneChip Mouse Genome 430 2.0 Array hybridization	GSM1350156
2 month old adult mice	wild type intestines	villi tissue collection	RNA extraction	Affymetrix GeneChip Mouse Genome 430 2.0 Array hybridization	GSM1350157
2 month old adult mice	wild type intestines	villi tissue collection	RNA extraction	Affymetrix GeneChip Mouse Genome 430 2.0 Array hybridization	GSM1350158
2 month old adult mice	wild type intestines	villi tissue collection	RNA extraction	Affymetrix GeneChip Mouse Genome 430 2.0 Array hybridization	GSM1350159
2 month old adult mice	wild type intestines	villi tissue collection	RNA extraction	Affymetrix GeneChip Mouse Genome 430 2.0 Array hybridization	GSM1350160
2 month old adult mice	Rb KO intestines	villi tissue collection	RNA extraction	Affymetrix GeneChip Mouse Genome 430 2.0 Array hybridization	GSM1350161
2 month old adult mice	Rb KO intestines	villi tissue collection	RNA extraction	Affymetrix GeneChip Mouse Genome 430 2.0 Array hybridization	GSM1350162
2 month old adult mice	Rb KO intestines	villi tissue collection	RNA extraction	Affymetrix GeneChip Mouse Genome 430 2.0 Array hybridization	GSM1350163
2 month old adult mice	Rb KO intestines	villi tissue collection	RNA extraction	Affymetrix GeneChip Mouse Genome 430 2.0 Array hybridization	GSM1350164
2 month old adult mice	Rb KO intestines	villi tissue collection	RNA extraction	Affymetrix GeneChip Mouse Genome 430 2.0 Array hybridization	GSM1350165
2 month old adult mice	Rb/Myc DKO intestines	villi tissue collection	RNA extraction	Affymetrix GeneChip Mouse Genome 430 2.0 Array hybridization	GSM1350166
2 month old adult mice	Rb/Myc DKO intestines	villi tissue collection	RNA extraction	Affymetrix GeneChip Mouse Genome 430 2.0 Array hybridization	GSM1350167
2 month old adult mice	Rb/Myc DKO intestines	villi tissue collection	RNA extraction	Affymetrix GeneChip Mouse Genome 430 2.0 Array hybridization	GSM1350168
2 month old adult mice	Rb/Myc DKO intestines	villi tissue collection	RNA extraction	Affymetrix GeneChip Mouse Genome 430 2.0 Array hybridization	GSM1350169
2 month old adult mice	Rb/Myc DKO intestines	villi tissue collection	RNA extraction	Affymetrix GeneChip Mouse Genome 430 2.0 Array hybridization	GSM1350170
2 month old adult mice	wild type intestines	crypts tissue collection	RNA extraction	Affymetrix GeneChip Mouse Genome 430 2.0 Array hybridization	GSM1350171
2 month old adult mice	wild type intestines	crypts tissue collection	RNA extraction	Affymetrix GeneChip Mouse Genome 430 2.0 Array hybridization	GSM1350172
2 month old adult mice	wild type intestines	crypts tissue collection	RNA extraction	Affymetrix GeneChip Mouse Genome 430 2.0 Array hybridization	GSM1350173
2 month old adult mice	wild type intestines	crypts tissue collection	RNA extraction	Affymetrix GeneChip Mouse Genome 430 2.0 Array hybridization	GSM1350174
2 month old adult mice	wild type intestines	crypts tissue collection	RNA extraction	Affymetrix GeneChip Mouse Genome 430 2.0 Array hybridization	GSM1350175
2 month old adult mice	Rb KO intestines	crypts tissue collection	RNA extraction	Affymetrix GeneChip Mouse Genome 430 2.0 Array hybridization	GSM1350176
2 month old adult mice	Rb KO intestines	crypts tissue collection	RNA extraction	Affymetrix GeneChip Mouse Genome 430 2.0 Array hybridization	GSM1350177
2 month old adult mice	Rb KO intestines	crypts tissue collection	RNA extraction	Affymetrix GeneChip Mouse Genome 430 2.0 Array hybridization	GSM1350178
2 month old adult mice	Rb KO intestines	crypts tissue collection	RNA extraction	Affymetrix GeneChip Mouse Genome 430 2.0 Array hybridization	GSM1350179
2 month old adult mice	Rb KO intestines	crypts tissue collection	RNA extraction	Affymetrix GeneChip Mouse Genome 430 2.0 Array hybridization	GSM1350180
2 month old adult mice	Rb/Myc DKO intestines	crypts tissue collection	RNA extraction	Affymetrix GeneChip Mouse Genome 430 2.0 Array hybridization	GSM1350181
2 month old adult mice	Rb/Myc DKO intestines	crypts tissue collection	RNA extraction	Affymetrix GeneChip Mouse Genome 430 2.0 Array hybridization	GSM1350182
2 month old adult mice	Rb/Myc DKO intestines	crypts tissue collection	RNA extraction	Affymetrix GeneChip Mouse Genome 430 2.0 Array hybridization	GSM1350183
2 month old adult mice	Rb/Myc DKO intestines	crypts tissue collection	RNA extraction	Affymetrix GeneChip Mouse Genome 430 2.0 Array hybridization	GSM1350184
2 month old adult mice	Rb/Myc DKO intestines	crypts tissue collection	RNA extraction	Affymetrix GeneChip Mouse Genome 430 2.0 Array hybridization	GSM1350185
2 month old adult mice	wild type intestines	crypts tissue collection	RNA extraction	Affymetrix GeneChip Mouse Genome 430 2.0 Array hybridization	GSM1350138
2 month old adult mice	wild type intestines	crypts tissue collection	RNA extraction	Affymetrix GeneChip Mouse Genome 430 2.0 Array hybridization	GSM1350139
2 month old adult mice	wild type intestines	crypts tissue collection	RNA extraction	Affymetrix GeneChip Mouse Genome 430 2.0 Array hybridization	GSM1350140
2 month old adult mice	wild type intestines	crypts tissue collection	RNA extraction	Affymetrix GeneChip Mouse Genome 430 2.0 Array hybridization	GSM1350141
2 month old adult mice	E2f TKO intestines	crypts tissue collection	RNA extraction	Affymetrix GeneChip Mouse Genome 430 2.0 Array hybridization	GSM1350142
2 month old adult mice	E2f TKO intestines	crypts tissue collection	RNA extraction	Affymetrix GeneChip Mouse Genome 430 2.0 Array hybridization	GSM1350143
2 month old adult mice	E2f TKO intestines	crypts tissue collection	RNA extraction	Affymetrix GeneChip Mouse Genome 430 2.0 Array hybridization	GSM1350144
2 month old adult mice	E2f TKO intestines	crypts tissue collection	RNA extraction	Affymetrix GeneChip Mouse Genome 430 2.0 Array hybridization	GSM1350145
2 month old adult mice	E2f TKO intestines	crypts tissue collection	RNA extraction	Affymetrix GeneChip Mouse Genome 430 2.0 Array hybridization	GSM1350146
2 month old adult mice	Myc KO intestines	crypts tissue collection	RNA extraction	Affymetrix GeneChip Mouse Genome 430 2.0 Array hybridization	GSM1350147
2 month old adult mice	Myc KO intestines	crypts tissue collection	RNA extraction	Affymetrix GeneChip Mouse Genome 430 2.0 Array hybridization	GSM1350148
2 month old adult mice	Myc KO intestines	crypts tissue collection	RNA extraction	Affymetrix GeneChip Mouse Genome 430 2.0 Array hybridization	GSM1350149
2 month old adult mice	Myc KO intestines	crypts tissue collection	RNA extraction	Affymetrix GeneChip Mouse Genome 430 2.0 Array hybridization	GSM1350150
2 month old adult mice	E2f/Myc QKO intestines	crypts tissue collection	RNA extraction	Affymetrix GeneChip Mouse Genome 430 2.0 Array hybridization	GSM1350151
2 month old adult mice	E2f/Myc QKO intestines	crypts tissue collection	RNA extraction	Affymetrix GeneChip Mouse Genome 430 2.0 Array hybridization	GSM1350152
2 month old adult mice	E2f/Myc QKO intestines	crypts tissue collection	RNA extraction	Affymetrix GeneChip Mouse Genome 430 2.0 Array hybridization	GSM1350153
2 month old adult mice	E2f/Myc QKO intestines	crypts tissue collection	RNA extraction	Affymetrix GeneChip Mouse Genome 430 2.0 Array hybridization	GSM1350154
2 month old adult mice	E2f/Myc QKO intestines	crypts tissue collection	RNA extraction	Affymetrix GeneChip Mouse Genome 430 2.0 Array hybridization	GSM1350155
2 month old adult mice	wild type intestines	villi tissue collection	ChIP-exo with E2F3 antibody	Next Generation Sequencing	GSM1606990
2 month old adult mice	Rb KO intestines	villi tissue collection	ChIP-exo with E2F3 antibody	Next Generation Sequencing	GSM1606986
2 month old adult mice	wild type intestines	crypts tissue collection	ChIP-exo with E2F3 antibody	Next Generation Sequencing	GSM1606988
2 month old adult mice	Rb KO intestines	crypts tissue collection	ChIP-exo with E2F3 antibody	Next Generation Sequencing	GSM1606984
2 month old adult mice	wild type intestines	villi tissue collection	ChIP-exo with MYC antibody	Next Generation Sequencing	GSM1606991
2 month old adult mice	Rb KO intestines	villi tissue collection	ChIP-exo with MYC antibody	Next Generation Sequencing	GSM1606987
2 month old adult mice	wild type intestines	crypts tissue collection	ChIP-exo with MYC antibody	Next Generation Sequencing	GSM1606989
2 month old adult mice	Rb KO intestines	crypts tissue collection	ChIP-exo with MYC antibody	Next Generation Sequencing	GSM1606985

**Table 2:** Reads mapping and peak calling metrics for ChIP-exo-seq.

**Samples**	**No. of mice**	**No. of total reads**	**% of duplicates**	**No. of uniquely mapped reads**	**No. of peak summits identified (GEM (FDR<0.01))**	**No. of peak summits identified (MACS2 (FDR<0.05))**	**No. of peak summits intersected (between GEM (FDR<0.01) and MACS2 (FDR<0.05))**
control_crypts_anti-E2f3	*n*=32	124,726,611	26.65%	81,486,676 (65.33%)	32,825	191,865	32,482
control_crypts_anti-Myc		112,880,763	42.41%	77,206,357 (68.40%)	159,282	783,127	158,997
control_villi_anti-E2f3	*n*=7	105,032,640	22.38%	65,370,657 (62.24%)	8,136	17,683	7,211
control_villi_anti-Myc		112,847,507	24.22%	70,997,848 (62.91%)	7,573	24,025	7,019
*RbKO*_crypts_anti-E2f3	*n*=27	111,728,323	20.10%	73,031,413 (65.37%)	28,139	80,247	27,200
*RbKO*_crypts_anti-Myc		89,929,148	24.04%	58,593,391 (65.16%)	32,545	71,705	31,244
*RbKO*_villi_anti-E2f3	*n*=7	69,199,986	20.67%	43,225,953 (62.47%)	15,549	41,624	14,668
*RbKO*_villi_anti-Myc		69,227,555	20.15%	43,699,768 (63.12%)	5,001	7,448	3,929

**Table 3:** Enrichment of tags (5 prime of reads) within peak regions

**Sample**	**totalTags**	**tagsInPeaks**	**peakBases**	**tagPerBaseInPeaks**	**tagPerBaseBackground**	**enrichRatio**
RbKO_crypts_anti-E2F3	73,031,413	505,408	767,088	0.66	0.03	23.80
RbKO_crypts_anti-Myc	58,593,391	378,701	879,116	0.43	0.02	19.38
RbKO_villi_anti-E2F3	43,225,953	190,358	415,604	0.46	0.02	27.88
RbKO_villi_anti-Myc	43,699,768	61,840	115,332	0.54	0.02	32.20
WT_crypts_anti-E2F3	81,486,676	595,603	914,648	0.65	0.03	21.09
WT_crypts_anti-Myc	77,206,357	2,874,150	4,457,068	0.64	0.03	22.69
WT_villi_anti-E2F3	65,370,657	117,363	207,340	0.57	0.02	22.73
WT_villi_anti-Myc	70,997,848	114,075	202,244	0.56	0.03	20.85
After shifting each read towards 3 prime for 14 bp, we calculated the Tags Per Base (TPB) across the genome for each sample. The tag means the 5 prime end of each read. tagPerBaseInPeaks=tagsInPeaks/peakBases, tagPerBaseBackground=(totalTags−tagsInPeaks)/(MouseGenomeSize - peakBases), enrichRatio=tagPerBaseInPeaks/tagPerBaseBackground.						
